# Hyper-prevalence of submicroscopic *Plasmodium falciparum* infections in a rural area of western Kenya with declining malaria cases

**DOI:** 10.1186/s12936-021-04012-6

**Published:** 2021-12-20

**Authors:** Kevin O. Ochwedo, Collince J. Omondi, Edwin O. Magomere, Julius O. Olumeh, Isaiah Debrah, Shirley A. Onyango, Pauline W. Orondo, Benyl M. Ondeto, Harrysone E. Atieli, Sidney O. Ogolla, John Githure, Antony C. A. Otieno, Andrew K. Githeko, James W. Kazura, Wolfgang R. Mukabana, Yan Guiyan

**Affiliations:** 1 Department of Biology, Faculty of Science and Technology, University of Nairobi, Nairobi, Kenya; 2 Sub-Saharan Africa International Centre for Excellence in Malaria Research, Homa Bay, Kenya; 3 Department of Biochemistry and Molecular Biology, Egerton University, Njoro, Kenya; 4 West Africa Centre for Cell Biology of Infectious Pathogen, Department of Biochemistry, Cell and Molecular Biology, University of Ghana, Accra, Ghana; 5 Centre for Global Health Research, Kenya Medical Research Institute, Kisumu, Kenya; 6 Centre for Global Health and Diseases, Case Western Reserve University, Cleveland, OH USA; 7 Program in Public Health, College of Health Sciences, University of California, Irvine, USA

**Keywords:** Submicroscopic, *Plasmodium* infection, Polymerase chain reaction, Blood smear, Western Kenya, Diagnostic tests

## Abstract

**Background:**

The gold standard for diagnosing *Plasmodium falciparum* infection is microscopic examination of Giemsa-stained peripheral blood smears. The effectiveness of this procedure for infection surveillance and malaria control may be limited by a relatively high parasitaemia detection threshold. Persons with microscopically undetectable infections may go untreated, contributing to ongoing transmission to mosquito vectors. The purpose of this study was to determine the magnitude and determinants of undiagnosed submicroscopic *P. falciparum* infections in a rural area of western Kenya.

**Methods:**

A health facility-based survey was conducted, and 367 patients seeking treatment for symptoms consistent with uncomplicated malaria in Homa Bay County were enrolled. The frequency of submicroscopic *P. falciparum* infection was measured by comparing the prevalence of infection based on light microscopic inspection of thick blood smears versus real-time polymerase chain reaction (RT-PCR) targeting *P. falciparum* 18S rRNA gene. Long-lasting insecticidal net (LLIN) use, participation in nocturnal outdoor activities, and gender were considered as potential determinants of submicroscopic infections.

**Results:**

Microscopic inspection of blood smears was positive for asexual *P. falciparum* parasites in 14.7% (54/367) of cases. All of these samples were confirmed by RT-PCR. 35.8% (112/313) of blood smear negative cases were positive by RT-PCR, i.e., submicroscopic infection, resulting in an overall prevalence by RT-PCR alone of 45.2% compared to 14.7% for blood smear alone. Females had a higher prevalence of submicroscopic infections (35.6% or 72 out of 202 individuals, 95% CI 28.9–42.3) compared to males (24.2%, 40 of 165 individuals, 95% CI 17.6–30.8). The risk of submicroscopic infections in LLIN users was about half that of non-LLIN users (OR = 0.59). There was no difference in the prevalence of submicroscopic infections of study participants who were active in nocturnal outdoor activities versus those who were not active (OR = 0.91). Patients who participated in nocturnal outdoor activities and use LLINs while indoors had a slightly higher risk of submicroscopic infection than those who did not use LLINs (OR = 1.48).

**Conclusion:**

Microscopic inspection of blood smears from persons with malaria symptoms for asexual stage *P. falciparum* should be supplemented by more sensitive diagnostic tests in order to reduce ongoing transmission of *P. falciparum* parasites to local mosquito vectors.

**Supplementary Information:**

The online version contains supplementary material available at 10.1186/s12936-021-04012-6.

## Background

Current vector control and parasite surveillance strategies have shown remarkable progress in reducing the global malaria burden [1]. In Kenya, malaria-endemic areas such as Homa Bay County have ongoing vector control intervention that include indoor residual spraying (IRS) and long-lasting insecticidal nets (LLINs) [2]. Malaria prevalence has decreased in these in these areas, largely as a result of these interventions [3]. There is concern, however, that these gains may not be durable because the exclusive use of microscopy for passive case detection of *Plasmodium falciparum* infection may not be sufficiently sensitive to detect submicroscopic infections [4]. A high prevalence of undetected submicroscopic malaria cases may contribute to a parasite reservoir that is sufficient to sustain ongoing *P. falciparum* transmission in endemic communities [5, 6].

Submicroscopic infections have been observed not only in high transmission settings but also in malaria endemic areas with seasonal or low transmission [7, 8]. These infections also show reduced parasite genetic diversity [9], fewer infective *Anopheles* [10, 11], lower adherence to anti-malarials drug regimens [12–14], and increased asexual parasite clearance rates [9, 15]. Previous studies of submicroscopic parasitaemia have primarily been concerned with its occurrence in pregnant women [16–20] and cross-sectional community surveys of asymptomatic individuals [3, 21–24]. However, fewer studies have focused on its occurrence as it pertains to malaria treatment-seeking behaviour [14, 25, 26]. The objective of this study was to determine the prevalence of submicroscopic infections among patients seeking malaria treatment at a rural health centre in western Kenya and the demographic and behavioural variables associated with these infections.

## Methods

### Study area and design

**Fig. 1:**
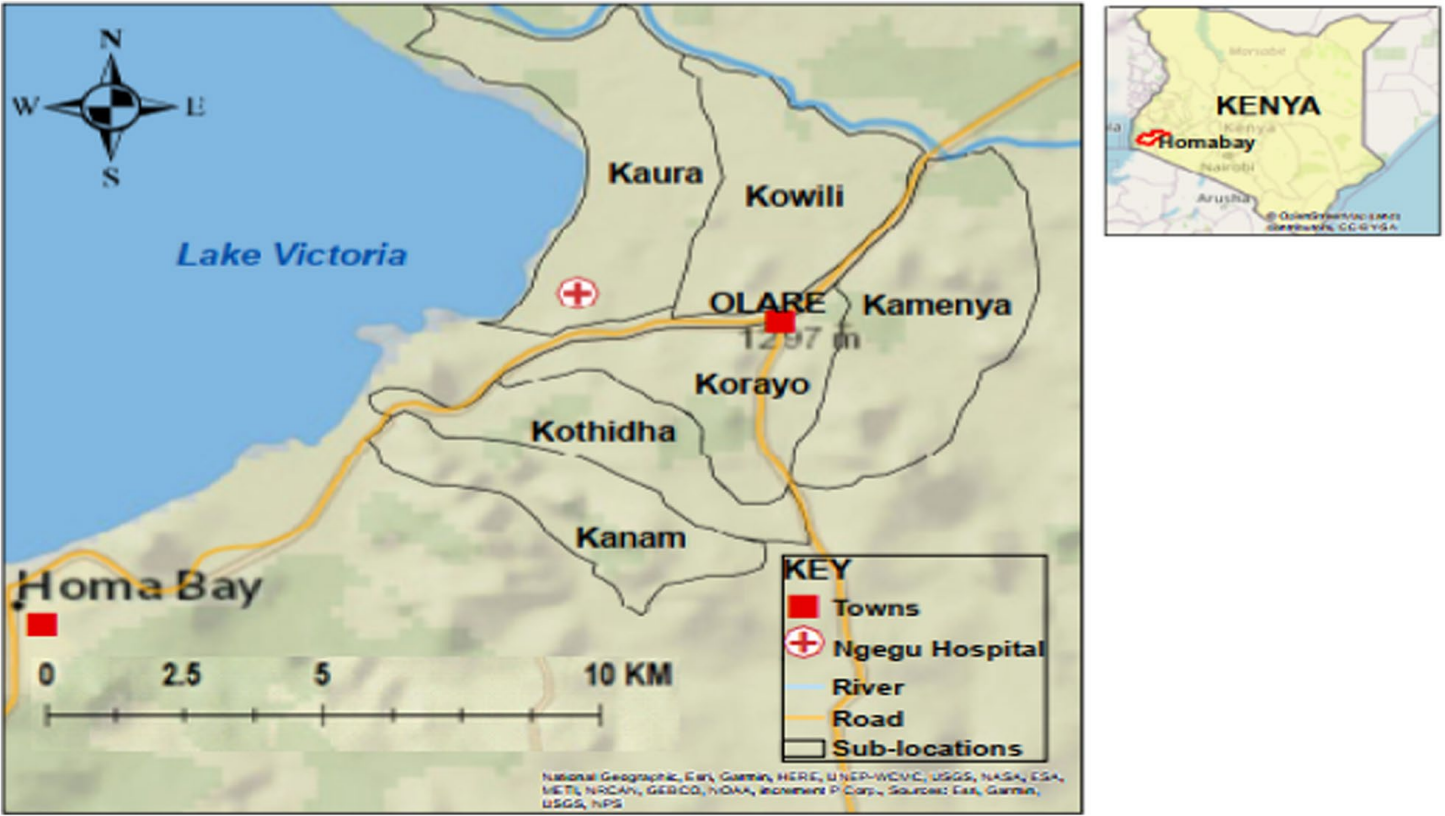
The study area map shows study sites (sublocations) in Homa Bay. The circle with the red cross represents Ngegu health facility, where patients from the six sublocations seek medical services

The study was conducted at the Ngegu health facility in Homa Bay County, western Kenya. This facility had a catchment population of 6,703 persons in 2020. The sampled patient population came from the Kochia location, which is divided into smaller administrative units referred as sub-locations. Study participants were residents of Kamenya, Kanam, Kaura, Korayo, Kothidha and Kowili sublocations located near the shore of Lake Victoria at a latitude of 34.64190E and 0.38000S with an elevation of 1143–1330 m above sea level (Fig. 1). The mean annual temperature is 22.7 °C. Rainfall is seasonal with two major peaks, March to May and October to December [27]. The study area is bordered by the Kimira-Oluch irrigation scheme, which has been demonstrated to have an impact on malaria transmission [28]. Homa Bay County is predominantly malaria-endemic, with approximately 20% overall *P. falciparum* infection prevalence [29]. The Ministry of Health has been conducting annual IRS with Actellic insecticide in the study area from February to March since 2018.

A health facility-based survey was performed, in which 367 patients seeking malaria treatment from six sub-locations were enrolled. Patient information such as LLIN ownership and use, occupation, and participation in nocturnal outdoor activities was collected. Nocturnal outdoor activities included casting fishing nets, setting and removing fishing traps, overnight fishing, early purchase of fish by small scale traders, farming, late evening trading at open-air markets, and waiting, picking up, and dropping off clients from motorcycle taxi riders (“Bodaboda”). Patient enrolment and data collection periods occurred in July and August of 2020, which coincided with the peak of *P. falciparum* transmission. The number of samples used was determined by the number of patients who sought malaria treatment and consented or assented to the study. Blood smear microscopy slides read at the hospital laboratory were confirmed by experienced microscopists at the Sub-Saharan Africa International Center of Excellence in Malaria Research (ICEMR) laboratory. All the samples were tested for submicroscopic infection by RT-PCR at the ICEMR laboratory at Tom Mboya University, Homa Bay.

### Processing of blood smears

Blood samples used in this study were obtained by antecubital venipuncture and immediately pipetted on filter paper and glass slides. Experienced hospital microscopists prepared and read Giemsa-stained slides for the presence and density of *Plasmodium* parasites. Slides for microscopic examination for the presence of *Plasmodium* parasites in patient blood samples were prepared and read by experienced hospital microscopists. Parasite density was estimated based on visualizing microscopic fields consisting of 200 leucocytes with the assumption of a standard value of 8,000 leucocytes per μL of blood. If no parasite was found after examining 200 fields at 100 × magnification, the microscopy result was declared negative. All slides were subjected to a second microscopy reading for quality control.

### DNA extraction and* Plasmodium falciparum* speciation

Genomic DNA was extracted from dried blood spots on filter paper following a modification of the Chelex resin (Chelex-100) saponin method [30]. *Plasmodium falciparum* species-specific 18S ribosomal RNA primers and probes were used to confirm the presence of parasite DNA [31]. PCR was run in a final volume of 12 µl containing 2 µL of parasite DNA, 6 µL of PerfeCTa® qPCR ToughMix™, Low ROX™ Master mix (2X), 0.5 µL of the species-specific probe, 0.4 µL of the forward species-specific primers (10 µM), 0.4 µL of the reverse species-specific primers (10 µM) and 0.1 µL of double-distilled water. The thermal profile used was 50 °C for 2 min, (95 °C for 2 min, 95 °C for 3 s and 58 °C for 30 s) for 45 cycles.

### Patient behavior, LLIN usage and other variables associated with submicroscopic infection

To assess the relationship between submicroscopic *P. falciparum* infection and LLIN use and participation in nocturnal outdoor activities, health centre study staff interviewed and completed a questionnaire for each participant. Information on LLIN ownership and usage, engagement in nocturnal outdoor activities, and occupations (i.e., student, non-Student (< 5 Children), farmer, trader, fishermen, motorcycle taxi riders, teacher, security, construction, unemployed and other) were collected. Only participants who acknowledged being outdoors from 1800 h–2000 h, 2000 h–2300 h, 2300 h–0400 h, and 0400 h–0600 h due to occupational requirements were considered involved in nocturnal outdoor activities.

### Statistical analysis

Patient data were entered into Microsoft Excel v. 2016 for cleaning and analysis. Descriptive statistics such as sum, mean, standard deviation, standard error and 95% confidence interval were used to summarize the population under study. Before comparison of mean value, data normality was confirmed using the Shapiro–Wilk normality test. To determine LLIN usage across gender, age and sub-location residence, multiple mean comparisons between these variables were performed using the Kruskal–Wallis test followed by Dunn’s multiple comparison test. Comparisons between blood smear positive and submicroscopic (RT-PCR positive, blood smear negative) groups were performed using Pearson chi-square. Binary logistic regression models were used to determine the association between microscopic and submicroscopic infections and potential determinants such as LLIN use, engagement in nocturnal outdoor activities and gender. A multivariate analysis was used to determine the relationship between the LLIN use, nocturnal outdoor activities, microscopic and submicroscopic infections. Analyses were performed in GraphPad Prism v.8.0.1 Software and SPSS version 25 for Windows. Data were considered statistically significant at p < 0.05.

## Results

### Patient characteristics

Study participant gender, age, LLIN usage, sublocation residence and participation in nocturnal outdoor activities are described in Table 1. More females than males participated in the study, and LLIN usage was greater among females than males (χ^2^ = 6.84, df 1, p = 0.009). Study participants were predominantly > 15 years (67%), while only 5% were < 5 years. LLIN use was significantly greater among children < 5 years relative to the 5–15 and > 15-year groups (Kruskal Wallis H test, *H *_*(2)*_ = 19.20, p < 0.0001). LLIN use was similar among participant sublocation residence as was nocturnal outdoor activity behaviour. Two study participants were pregnant. Male patients participating in nocturnal outdoor activities were 68% (57/84) whereas females were 32% (27/84). There was no significant difference in LLIN use between participants who engaged in nocturnal outdoor activities and used LLIN while indoors versus non-LLIN users who participated in nocturnal outdoor activities (χ^2^ = 2.97, df1, p = 0.085).

**Table 1:** Characteristics of study participants including the usage of long-lasting insecticide-treated nets and participation in nocturnal outdoor activities

Parameter		N	Bed net usage n (%)	P-value
Name	Level			
Gender	Female	202	106 (52.48)	0.009
	Male	165	64 (38.79)	
Age group	< 5	19	18 (94.74)	< 0.0001
	5–15	102	42 (41.18)	
	≥ 15	246	110 (44.72)	
Sub-location	Kamenya	60	29 (48.33)	0.4391
	Kanam	78	35 (44.87)	
	Kaura	111	48 (43.24)	
	Korayo	59	24 (40.68)	
	Kothidha	23	12 (52.17)	
	Kowili	36	22 (61.11)	
Nocturnal outdoor activities	Yes	84	32 (38.10)	0.085
	No	283	138 (48.76)	

N represents the total number of individuals while n represents the cases. Only patients who stated that they were outdoor from 1800 h–2000 h, 2000 h–2300 h, 2300 h–0400 h, and 0400 h–0600 h were considered to be engaged in nocturnal outdoor activities

### Prevalence of microscopy detectable and submicroscopic *P. falciparum* infections

Asexual stage malaria parasites were found in 54 (15%) of the 367 microscopically-screened blood smears. The prevalence of microscopy positive infections in males (17%) was slightly higher than that of females (13%). Children < 5 years had the lowest infection rate by microscopy (11%), while those aged 5–15 had the highest infection rates (27%), followed by adults (10%). On the basis of parasite density, children < 5 years, those age group 5–15 years, and adults recorded 219, 957 and 330 asexual parasites per microlitre of whole blood, respectively. All the slide positive results were confirmed for asexual parasite DNA by RT-PCR.

Thirty-six percent (112/313) of the slide negative samples were found to be *P. falciparum* positive by RT-PCR and labelled as submicroscopic. These infections accounted for 67% (112/166) of confirmed malaria cases. Detection of submicroscopic infections (31%) were significantly greater than that of microscopic infections (15%) (χ^2^ = 27.81, df 1, P < 0.0001). Female study participants had 36% (72/202, 95%, CI: 28.9–42.3) more submicroscopic infections than males 24% (40/165, 95%, CI: 17.6–30.8). Female patients had a significant difference between microscopic and submicroscopic infections (χ^2^ = 4.39, df 1, p = 0.036), but males did not. Neither of two pregnant study participants had microscopy positive infection; one had a submicroscopic infection. The prevalence of submicroscopic infection was highest in children < 5 years (42%), followed by adults (30%) and children aged 5–15 years (29%). Only one of eight male patients < 5 years had submicroscopic infections, compared to seven out of 11 females of the same age.

There were more submicroscopic infections than microscopic infections at the sub-location level. Kaura had the highest prevalence of *P. falciparum* (both microscopic and submicroscopic) infections (51.3%), followed by Korayo (45.7%), Kanam (44.8%), Kamenya (43.3%), Kothidha (39.1%) and Kowili (33.3%). Korayo had the highest percentage of submicroscopic infections, 38.9% (23/59, 95%, CI: 26.1–51.8), followed by Kamenya (35%, 21/60, 95%, CI: 22.5–47.4), Kaura (27.9%, 31/111, 95%, CI: 19.4–36.4), Kowili (27.78%, 10/36, 95%, CI: 12.4–43.1), Kanam (26.9%, 21/78, 95%, CI: 16.8–36.9) and Kothidha (26.1%, 6/23, 95%, CI: 6.67–45.5). The Kruskal–Wallis H test revealed a significant difference between microscopic and submicroscopic infections across all sublocations (*H*_*(11)*_ = 41.21, p > 0.001). Moreover, Dunn’s test pairwise comparison showed a significant difference between microscopic and submicroscopic infections in Kamenya (p = 0.0320) and Korayo (p = 0.0019).

### Relationship between submicroscopic infections, patient behaviour and LLIN usage

The odds of females seeking malaria treatment having submicroscopic infections was 1.73 higher than males (Table 2). Use of LLIN had a significant effect on microscopic and submicroscopic infections (F (2, 362) = 3.029, p = 0.05; Wilk’s = 0.984, partial η^2^ = 0.016) (Additional file 1: Table S1). However, when considered separately, the use of LLIN had a significant effect on microscopic infections (F (1, 363) = 4.499, p = 0.035; partial η^2^ = 0.012) but not on submicroscopic infections (Additional file 1: Table S2). Interestingly, patients using LLINs had a significantly higher prevalence of submicroscopic infections (36.5%) (χ^2^ = 5.29, df 1, p = 0.021) than non-users (25.4%). This implies that the chance of LLIN users harbouring submicroscopic infections was about half that of non-LLIN users (OR = 0.59). Though not significant (χ^2^ = 0.136, df 1, p = 0.713), more patients involved in nocturnal outdoor activities had submicroscopic infections (32.1%) compared to those restricted indoors during night hours (25.3%). Participants who engaged in nocturnal outdoor activities were 0.91 times more likely to have submicroscopic infections than those who stayed indoors (Table 2). This, however, varied when comparison was made between patients engaged in nocturnal outdoor activities but using LLINs while indoors versus non-LLIN users involved in nocturnal outdoor activities. Those LLIN users who participated in nocturnal outdoor activities were 1.48 times more likely to have submicroscopic infections and have high infections (37.5%) than non-LLINs users involved in nocturnal outdoor activities (28.85%) (χ^2^ = 0.68, df 1, p = 0.41).

**Table 2:** Socioeconomic and behavioural determinants of malaria infections

Parameter		N	Microscopic infections			N	Submicroscopic infections		
Name	Level		n (%)	OR (95%, CI)	P-value		n (%)	OR (95%, CI)	P-value
Gender	Female	202^**a**^	26 (12.87)	0.72 (0.41–1.29)	0.272	202^**a**^	72 (35.64)	1.73 (1.10–2.74)	0.019
	Male	165	28 (16.97)			165	40 (24.24)		
LLIN usage	Yes	170	19 (11.18)	1.72 (0.94–3.13)	0.078	170	62 (36.47)	0.59 (0.38–0.93)	0.022
	No	197^**a**^	35 (17.77)			197^**a**^	50 (25.38)		
Nocturnal outdoor activities*	Yes	84	10 (11.90)	1.36 (0.65–2.84)	0.409	84	27 (32.14)	0.91 (0.54–1.53)	0.713
	No	283^**a**^	44 (15.55)			283^**a**^	85 (30.04)		
Nocturnal outdoor activities^⁑^	LLIN usage	32	1 (3.13)	6.49 (0.78–53.89)	0.083	32^**a**^	12 (37.50)	1.48 (0.58–3.77)	0.411
	No LLIN usage	52^**a**^	9 (17.31)			52	15 (28.85)		

N represents total number of individuals while n represents the cases. The asterisk (*) represent general effect of nocturnal outdoor activities on microscopic and submicroscopic infections while the vertical double asterisk (^⁑^) represents interaction effects between nocturnal outdoor activities and LLIN usage. Small letter “**a**” refers to reference category

## Discussion

In this study age, gender and use of LLINs were factors in the occurrence of submicroscopic *P. falciparum* infections in patients with malaria symptoms seeking treatment in Homa Bay County's six sub-locations. The use of LLINs and being female was linked to a high prevalence of submicroscopic infections. Interestingly, the effect of LLIN usage on submicroscopic infection prevalence was also observed among users who participated in nocturnal outdoor activities. Furthermore, this study was unable to establish a conclusively link between the observed microscopic *P. falciparum* infections and outdoor transmission. This is due to the fact that only one LLIN user participating in nocturnal outdoor activities had microscopic infections, compared to nine infected non-LLIN users engaged in nocturnal outdoor activities.

The study findings revealed that male patients were more likely than females to test positive for microscopic infections. This could be attributed to existing social behavioural differences, such as more males engaging in nocturnal outdoor activities, which kept them out of the intervention coverage and low LLIN use when compared to female patients. Low LLIN use and participation in activities outside of intervention coverage [32–34] have been demonstrated to increase exposure to biting by infected female Anophelines [25, 35, 36]. As a result, this study findings corroborate previous research that reported a high prevalence of *P. falciparum* slide positivity in males [37, 38]. The high levels of microscopic *P. falciparum* infections and parasite density in the 5–15 age group were linked to a low number of LLIN use thus predisposed to bites from infected malaria vectors [25, 39, 40]. Children < 5 years were less likely to have microscopic infections because the majority used LLINs. As previously reported [41, 42], the low proportion of microscopic infections among this age group, as well as the high use of LLINs, was a good indicator of strict parental care. Patients who participated in nocturnal outdoor activities were more likely to have microscopic infections than patients who stayed indoors. However, patients who engaged in nocturnal outdoor activities and did not use LLINs while indoors were at a higher risk. These findings imply that biting by infected *Anopheles* mosquitoes occurs at the transition point from LLINs coverage to outdoor or vice versa. With these findings, the study suggests that outdoor malaria transmission is low in the six sub-locations. This finding supports previous findings in 2018 and 2019 by Ondeto et al., (pers. commun.) on an increased population of *Anopheles arabiensis* collected outdoors, with blood meal results indicating a feeding preference for bovines in the study area.

Despite the microscopic prevalence of 14.7%, there were a large number of clinically positive cases that were missed because they were submicroscopic infections. The high levels of undetected infections may have a significant impact on existing malaria intervention strategies, potentially resulting in a plateauing of malaria cases in the future. The high number of missed cases is attributed to declining malaria prevalence, which has resulted in the area transitioning to a low malaria transmission zone [3]. Additionally, these low transmission zones have been reported to be prone to submicroscopic *P. falciparum* infections [8]. The reduced microscopic infections rates and increased levels of submicroscopic infections within this study site may indicate declining parasite genetics [9], host exposure to fewer bites by infected Anophelines [10, 11], and a faster rate of acquired immunity acquisition due to fewer parasite clones [15, 43]. These three underlining factors have been previously linked to increased levels of submicroscopic *P. falciparum* infections.

With an odds ratio of 1:8 for detecting submicroscopic infection in males versus females, these observations were slightly higher than previously reported ratio of 1:4 in patients from low transmission zones within Belaga district of Malaysia [24]. The high levels of submicroscopic infections in female patients support findings that females have a higher rate of asexual stage parasite clearance than males [44]. Although few in numbers, children < 5 years had the highest percentage of submicroscopic infections than the rest of age groups. The high number of adults infected with submicroscopic infections is consistence with the findings of a systematic review on drivers of these infections, which found age to be a significant determinant [45]. These high levels of submicroscopic infections in adults could be attributed to acquired immunity that suppresses parasite load, self-prescription, poor adherence to anti-malarial drug regimens, and a high prevalence of recently acquired infections [12, 13]. Patients living in Kaura sublocation had the highest malaria burden of any of the six sub-locations studied. This sublocation is located along the shores of Lake Victoria, which could be a confounding factor in the observed periodic prevalence. Sublocations with low LLIN use had a higher prevalence of both microscopic and submicroscopic infections than those with high LLIN use.

In contrast to the previously observed link between LLIN use and microscopic infections, patients who used LLINs were more likely to have submicroscopic infections. When the study was narrowed down to establish the link between nocturnal outdoor activities, LLIN use, and submicroscopic infections, a similar observation was made. In general, patients who participated in nocturnal outdoor activities had a higher chance of having submicroscopic infections than those who stayed indoors. This was largely influenced by the high LLIN usage among those involved in nocturnal outdoor activities. These findings demonstrates that an increase in malaria vector interventions may be directly or indirectly related to an increase in submicroscopic infections in this study site. Perhaps the interventions are limiting human-vector contact, lowering both biting and sporozoite inoculation rates, implying that low biting rates are a cause of rising submicroscopic infections [10, 11]. Additionally, the influence of LLIN integrity cannot be overlooked as the study did not consider this. Reduced LLIN integrity confers partial protection to the host against bites by infected malaria vectors [46].

Despite continued IRS, LLIN use, and lower periodic malaria prevalence in the six study sublocations, submicroscopic infections persisted in this rural area of western Kenya. As a result, the accumulation of undetected and untreated infections may continue to stymie efforts to achieve long-term malaria elimination. Further, female *Anopheles* have been successfully infected by submicroscopic infections [5, 6]. With rapid diagnostic kits clearly playing a significant role in malaria monitoring during the COVID-19 era in most countries [47], this study suggests supplementing microscopy with ultrasensitive malaria Rapid Diagnostic Tests (or PCR in areas where this is feasible) targeting patients with malaria-like symptoms.

## Conclusion

In the six study sublocations in Homa Bay County, this study found a high prevalence of submicroscopic infections, resulting in a large number of undetected and untreated patients who may serve as a reservoir for continuing transmission of *P. falciparum*. This result suggests that combined diagnosis using microscopy in conjunction ultrasensitive Rapid Diagnostic Tests or PCR is appropriate in areas with low *P. falciparum* transmission.

## Supplementary Information

**Additional file 1: Table S1.** Multivariate tests. **Table S2.** Test of between-subject effects.

## Data Availability

The dataset used in this study is available from the corresponding author upon request.

## Abbreviations

RT-PCR: Real-time polymerase chain reaction
LLIN: Long-lasting insecticide-treated net
CI: Confidence interval
OR: Odds ratio
IRS: Indoor residual spraying
DBS: Dried blood spots
DNA: Deoxyribonucleic acid
PCR: Polymerase chain reaction
