# Supplementary material for: Hyper-prevalence of submicroscopic Plasmodium falciparum infections in a rural area of western Kenya with declining malaria cases
Source: Malar J. 2021 Dec 20;20:472. doi: 10.1186/s12936-021-04012-6 (PMC8685826; doi:10.1186/s12936-021-04012-6)
Supplement: Supplementary file 1 — Additional file 1: Table S1. Multivariate tests. Table S2. Test of between-subject effects. [file 12936_2021_4012_MOESM1_ESM.docx]

**S1 Table:**

| **Effect** | | **Value** | **F** | **Hypothesis df** | **Error df** | **Significance** | **Observed Power^c^** |
| --- | --- | --- | --- | --- | --- | --- | --- |
| Intercept | Pillai's Trace | 0.353 | 98.600^b^ | 2 | 362 | 0.000 | 1.000 |
|  | Wilks' Lambda | 0.647 | 98.600^b^ | 2 | 362 | 0.000 | 1.000 |
|  | Hotelling's Trace | 0.545 | 98.600^b^ | 2 | 362 | 0.000 | 1.000 |
|  | Roy's Largest Root | 0.545 | 98.600^b^ | 2 | 362 | 0.000 | 1.000 |
| Net usage | Pillai's Trace | 0.016 | 3.029^b^ | 2 | 362 | 0.050 | 0.585 |
|  | Wilks' Lambda | 0.984 | 3.029^b^ | 2 | 362 | 0.050 | 0.585 |
|  | Hotelling's Trace | 0.017 | 3.029^b^ | 2 | 362 | 0.050 | 0.585 |
|  | Roy's Largest Root | 0.017 | 3.029^b^ | 2 | 362 | 0.050 | 0.585 |
| Outdoor activities | Pillai's Trace | 0.004 | 0.707^b^ | 2 | 362 | 0.494 | 0.169 |
|  | Wilks' Lambda | 0.996 | 0.707^b^ | 2 | 362 | 0.494 | 0.169 |
|  | Hotelling's Trace | 0.004 | 0.707^b^ | 2 | 362 | 0.494 | 0.169 |
|  | Roy's Largest Root | 0.004 | 0.707^b^ | 2 | 362 | 0.494 | 0.169 |
| Net usage * Outdoor activities | Pillai's Trace | 0.004 | 0.708^b^ | 2 | 362 | 0.493 | 0.169 |
|  | Wilks' Lambda | 0.996 | 0.708^b^ | 2 | 362 | 0.493 | 0.169 |
|  | Hotelling's Trace | 0.004 | 0.708^b^ | 2 | 362 | 0.493 | 0.169 |
|  | Roy's Largest Root | 0.004 | 0.708^b^ | 2 | 362 | 0.493 | 0.169 |
| a. Design: Intercept + Net usage + Outdoor activities + Net usage * Outdoor activities | | | | | | | |
| b. F-value | | | | | | | |
| c. Computed using alpha ≤0.05 | | | | | | | |

**S2 Table.**

| **Source** | **Dependent Variable** | **Type III Sum of Squares** | **df** | **Mean Square** | **F** | **Sig.** | **Partial Eta Squared** | **Noncent. Parameter** | **Observed Power^c^** |
| --- | --- | --- | --- | --- | --- | --- | --- | --- | --- |
| Corrected Model | Microscopy Results | 0.653^a^ | 3 | 0.218 | 1.741 | 0.158 | 0.014 | 5.224 | 0.454 |
|  | Sub-Microscopy | 1.211^b^ | 3 | 0.404 | 1.913 | 0.127 | 0.016 | 5.740 | 0.494 |
| Intercept | Microscopy Results | 4.089 | 1 | 4.089 | 32.696 | 0.000 | 0.083 | 32.696 | 1.000 |
|  | Sub-Microscopy | 24.847 | 1 | 24.847 | 117.733 | 0.000 | 0.245 | 117.733 | 1.000 |
| Net usage | Microscopy Results | 0.563 | 1 | 0.563 | 4.499 | 0.035 | 0.012 | 4.499 | 0.562 |
|  | Sub-Microscopy | 0.666 | 1 | 0.666 | 3.156 | 0.076 | 0.009 | 3.156 | 0.426 |
| Outdoor activities | Microscopy Results | 0.172 | 1 | 0.172 | 1.375 | 0.242 | 0.004 | 1.375 | 0.215 |
|  | Sub-Microscopy | 0.055 | 1 | 0.055 | 0.262 | 0.609 | 0.001 | 0.262 | 0.080 |
| Net usage * Outdoor activities | Microscopy Results | 0.134 | 1 | 0.134 | 1.069 | 0.302 | 0.003 | 1.069 | 0.178 |
|  | Sub-Microscopy | 0.018 | 1 | 0.018 | 0.087 | 0.768 | 0.000 | 0.087 | 0.060 |
| Error | Microscopy Results | 45.401 | 363 | 0.125 |  |  |  |  |  |
|  | Sub-Microscopy | 76.609 | 363 | 0.211 |  |  |  |  |  |
| Total | Microscopy Results | 54.000 | 367 |  |  |  |  |  |  |
|  | Sub-Microscopy | 112.000 | 367 |  |  |  |  |  |  |
| Corrected Total | Microscopy Results | 46.054 | 366 |  |  |  |  |  |  |
|  | Sub-Microscopy | 77.820 | 366 |  |  |  |  |  |  |
| a. R Squared = .014 (Adjusted R Squared = .006) | | | | | | | | | |
| b. R Squared = .016 (Adjusted R Squared = .007) | | | | | | | | | |
| c. Computed using alpha ≤ 0.05 | | | | | | | | | |
